# The use of technology as environmental enrichment in zoos: A scoping review

**DOI:** 10.1017/awf.2025.10038

**Published:** 2025-11-04

**Authors:** Lesia Hryhorenko, Todd McWhorter, Alexandra Whittaker, Eduardo J. Fernandez

**Affiliations:** School of Animal and Veterinary Sciences, The University of Adelaide, Roseworthy, SA 5371 Australia

**Keywords:** Animal-Computer Interaction (ACI), animal welfare, enrichment, interactive, technology, zoo

## Abstract

Technological enrichment, such as motion sensors, touchscreens, and response-independent feeders, offer innovative ways to enhance animal welfare in captivity by promoting species-appropriate behaviours and cognitive stimulation. A scoping review of 22 publications comprising 25 studies identified various technologies, with computers being the most common, and sensory enrichment the most frequent type implemented. Positive or neutral welfare outcomes were common, though some negative effects were also reported. Primates and carnivores were the most frequently studied groups. Despite increasing research since 2012, gaps remain, including limited peer-reviewed studies and a need for standardised methodologies to better evaluate the impact of technological enrichment.

## Introduction

The welfare of animals in zoos, aquaria, and wildlife parks is a core concern, with environmental enrichment being a key strategy for promoting well-being. Environmental enrichment refers to modifications to the environment that encourage species-appropriate behaviours (Young 2013; Fernandez 2022). Common forms include social, sensory, structural, food-based, and cognitive enrichment, all aimed at promoting behavioural diversity and reducing behaviours associated with negative welfare, such as stereotypies (Shyne 2006; de Azevedo *et al.* 2007; Hoy *et al.* 2010; Clay & Visseren-Hamakers 2022). Traditional enrichment techniques, such as puzzle feeders, novel objects, sensory stimuli, and food variation, have shown benefits including reduced stress and improved health (Shyne 2006; Fernandez & Martin 2021; Weibel *et al.* 2021; dos Santos Lemes Lechuga *et al.* 2023).

The advent of technological enrichment offers novel ways to enhance animal welfare through dynamic, automated devices. Technological enrichment involves automated mechanical devices, whether interactive or non-interactive (for operational definition, see *Materials and methods*). Such technologies include motion-sensing projectors, touchscreens, response-independent feeders, speakers, and video-based stimuli (Clay *et al.* 2011; Ogura 2012; Damasceno *et al.* 2017; Calapai *et al.* 2023; Grant *et al.* 2023). Examples include Markowitz *et al.*’s (1995) introduction of motion-sensor speakers that resulted in food rewards if followed by an African leopard (*Panthera pardus*), Carlstead *et al.*’s (1991) use of a response-independent feeder with a black bear (*Ursus americanus*), and more recently, Perdue’s (2016) provision of touchscreen tablets for interactive tasks with sun bears (*Helarctos malayanus*). Although early studies suggested potential for technological enrichment to positively influence animal behaviour and welfare, research on technological enrichment is still limited (Carter *et al.* 2021; Kresnye *et al.* 2022).

Related fields such as Animal-Computer Interaction (ACI) and Animal-Centred Computing (ACC) have also contributed to the development of animal-facing technologies, often focusing on interface design, usability, or species agency (French *et al.* 2019, 2021, 2022; Coe & Hoy 2020). However, many ACI/ACC studies do not assess behavioural changes or welfare impacts in a comparative framework, which limits their direct applicability to welfare-focused enrichment evaluation. This review therefore focuses specifically on technological enrichment studies that assess behavioural outcomes across at least two conditions (e.g. baseline vs enrichment), to evaluate their potential for improving animal welfare in zoo settings.

The effectiveness of technological enrichment is influenced by several factors, including the type of technology used, species-specific preferences, and the role of interactivity. Interactivity has become a key area of debate. Some researchers argue that interactive experiences, where animals can influence the outcome and incorporate choice and control over the stimuli or events involved, are vital for cognitive stimulation and behavioural flexibility (Englund & Cronin 2023; Rust *et al.* 2024). Others suggest that passive technologies, where animals do not control the stimuli, can also provide valuable enrichment (de Azevedo *et al.* 2007; Clay *et al.* 2011; Ogura 2012). Understanding the impact of interactivity on welfare outcomes is essential to optimise these technological tools.

The growing interest in technological enrichment is partly driven by advancements in technology that have made some forms, such as motion sensors and audio systems, more accessible and affordable. However, interactive systems, such as touchscreen interfaces and customised projections remain costly and require specialised infrastructure, limiting their widespread adoption in zoos and aquaria. Despite these limitations, the increasing availability of certain technologies continues to create new opportunities for dynamic enrichment that were previously unavailable (Clay *et al.* 2011). This is especially important as our understanding of the needs of captive animals continues to grow, highlighting the necessity of refining enrichment strategies to better support their welfare. Technological tools can complement or enhance conventional enrichment methods, offering fresh opportunities to engage animals in ways that better mirror their natural behaviours.

Despite its promise, technological enrichment faces challenges, such as high costs, technical failures, and uncertainty regarding effectiveness and safety (Clay *et al.* 2011; Scheer *et al.* 2019). Existing studies have yielded mixed results, with some reporting positive impacts on behaviour and welfare (Carlstead *et al.* 1991; Clay *et al.* 2011), while others indicate neutral (Perdue *et al.* 2012; Carter *et al.* 2021) or negative effects (Kim-McCormack *et al.* 2016). A detailed review of existing research is needed to better understand how best to tailor technological enrichment to meet the specific needs of species in zoo environments.

The role of technology as an enrichment tool in zoo settings remains an underexplored area, despite growing interest in its potential to enhance animal welfare. Previous reviews, such as Kresnye *et al.* (2022), have focused upon the integration of technology but have not considered it as the primary form of enrichment. This review aims to address this gap by systematically identifying and synthesising studies that examined technological enrichment as a primary intervention, with direct behavioural outcomes compared across conditions (e.g. pre/post, with/without enrichment). By focusing on studies that isolated the effects of technological enrichment through two-condition comparisons, this review provides a rigorous assessment of both welfare outcomes and methodological approaches. Specifically, we examine: (1) the types of technologies used; (2) the behavioural impacts observed; (3) the role of interactivity; and (4) how outcomes were measured.

## Materials and methods

This scoping review was conducted in accordance with the Joanna Briggs Institute (JBI) methodology for scoping reviews (Peters *et al.* 2020). The scoping review is reported using the PRISMA extension for scoping reviews (PRISMA-ScR) framework (Tricco *et al.* 2018).

### Search strategy

The search strategy aimed to locate published articles, using a three-step approach. First, an initial limited search of Web of Science (including CAB Abstracts) and ScienceDirect was conducted to identify articles on the topic. The titles, abstracts, and index terms of relevant articles were examined to develop a comprehensive search strategy for additional databases: Scopus and Zoological Record. The search strategy was adapted for each database with the help of the university’s librarian, and included keywords and index terms related to zoos, aquaria, wildlife parks, enrichment, and technology. These database searches occurred from August to September of 2024. The reference lists of included studies were screened to identify additional relevant articles. The language was limited to English, and studies published from the inception of each database were included to capture the development of technological enrichment over time, e.g. search term for Web of Science (Zoological Record): (Zoological gardens OR Zoos OR Aquaria OR Aviaries OR Wildlife parks OR Care in captivity [Subject Descriptors] AND enrich OR enrichment OR behavioural enrichment [Topic] AND build OR built OR digital OR compose OR mechanical OR computer OR electromechanical OR technological OR device OR system OR create OR touch OR display OR hardware OR design OR sensor OR push OR button OR animal-computer interaction [Topic] AND Article [Document Types]).

### Publication selection

**Figure 1.**
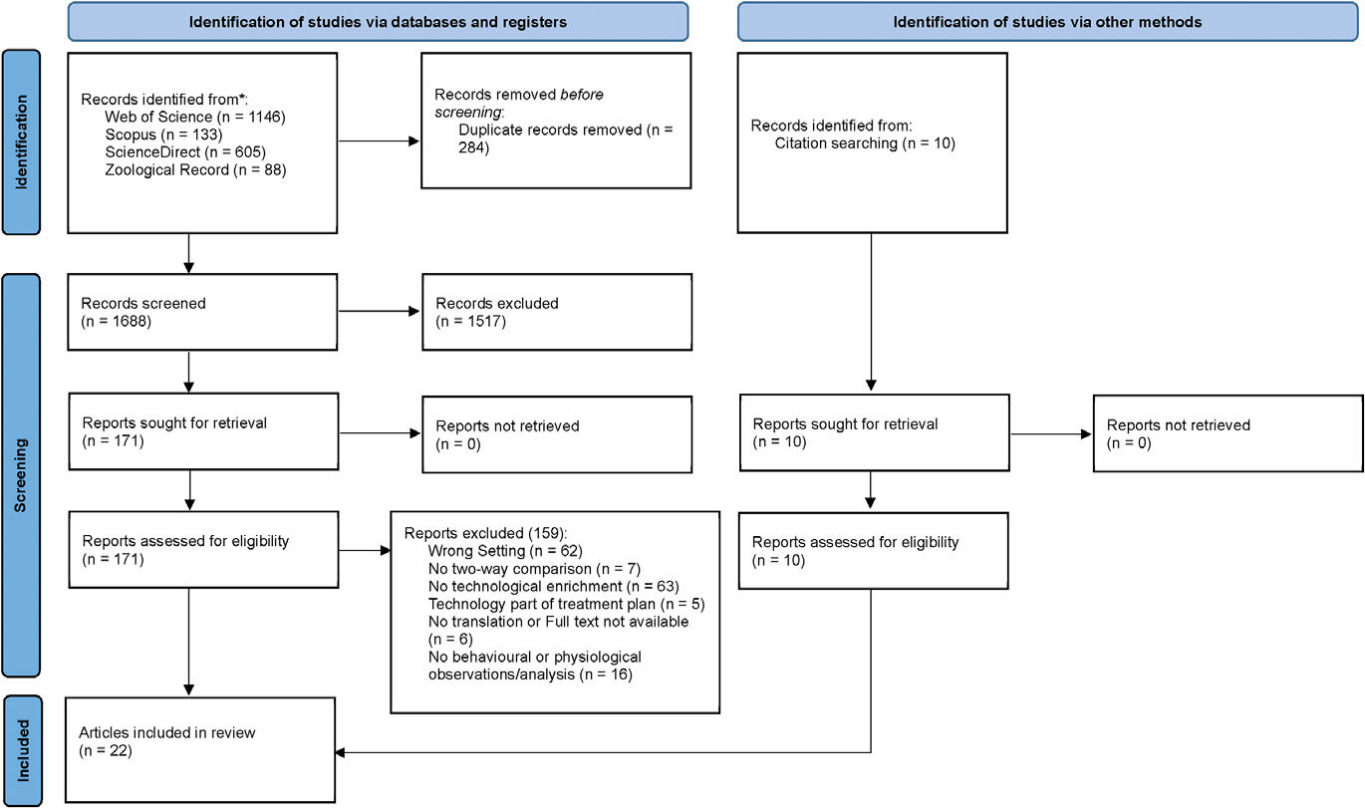
PRISMA flow diagram showing the process of identifying and selecting studies for inclusion in the scoping review on technological enrichment for animals in zoos, aquaria, and wildlife parks. The diagram summarises records from four databases (Web of Science, Scopus, ScienceDirect, Zoological Record) and citation searching. A total of 1,688 records were screened, 181 full-text articles assessed for eligibility, and 22 articles (comprising 25 studies) included in the final review.

All identified citations were imported into Covidence (Veritas Health Innovation, Melbourne, VIC, Australia), where duplicate records were automatically removed. Titles and abstracts were screened by a single reviewer against the inclusion criteria. Papers were excluded if they were not published in English, were not peer-reviewed, did not focus on technological enrichment, or were conducted outside of zoos, aquaria, or wildlife parks. Full-text articles of potentially relevant research were retrieved and assessed by two independent reviewers to ensure adherence to the inclusion criteria. Any disagreements during the selection process were resolved through discussion or consultation with a third reviewer. Reasons for the exclusion of studies during full-text screening were documented and reported in the final review. The study selection process was reported using a PRISMA flow diagram (Figure 1).

### Eligibility criteria

Publications were included if they were conducted in zoos, aquaria, or wildlife parks (a primarily outdoor zoological facility typically focused on native or regional species), focused on non-human animals, and investigated technological enrichment. Technological enrichment was defined as devices or events with automated internal mechanics that are at least partially self-controlled, such as touchscreens, audio systems, response-independent feeders, and motion-activated devices. In addition, technological enrichment could be classified as interactive or non-interactive, with ‘interactive’ defined as manipulation of the device or event that results in a direct consequence as a result of the interaction (e.g. change of screen or food delivered). Papers needed to provide measurable behavioural outcomes, such as reductions in abnormal behaviours, increased activity, or enhanced behavioural diversity, with outcomes classified as positive, neutral, or negative. Studies were included if they featured a two-way comparison (i.e. at least two conditions compared), such as pre/post comparisons, enrichment versus no enrichment, or comparisons between different types of enrichment. This ensured that technological enrichment was directly evaluated against another condition rather than as part of a broader enrichment package or no comparison at all.

Papers were excluded if technological enrichment was assessed within a multi-enrichment ‘treatment package’ where multiple enrichment types (e.g. food-based and technological enrichment together) were introduced simultaneously but without a direct comparison that isolated the effect of the technological component. For example, Hunter *et al.* (2002) provided harbour (*Phoca vitulina*) and grey (*Halichoerus grypus*) seals with a combination of enrichment items — including a bubble machine, artificial kelp, and toys — but evaluated behavioural outcomes in response to the entire package rather than distinguishing the effect of the bubble-based enrichment. Conversely, studies like Caselli *et al.* (2022) were included because each enrichment type, including the technological component (a speaker-based auditory enrichment), was analysed separately using appropriate comparison conditions.

Additionally, papers were excluded if they involved grey literature or if enrichment devices were non-technological and required direct animal manipulation, such as a puzzle feeder.

This strict inclusion structure was necessary to ensure that technological enrichment was evaluated as the primary independent variable. Studies where technology was used alongside multiple enrichment types (e.g. food and social enrichment) or where no behavioural comparison was present were excluded to maintain the review’s focus on outcomes directly attributable to the technological component. This framework supports valid cross-study comparison of behavioural impacts.

### Data extraction

Data were extracted from each included publication by two independent reviewers using a standardised data extraction tool (Covidence), that was then exported to Excel® for refinement. The extracted data included details about the authors, publication year, publication title, journal, country, setting (zoo, aquarium, or wildlife park), species studied, sample size, enrichment type, type of technology, interactivity (interactive or non-interactive), behavioural outcomes, and methods of behavioural assessment.

Each publication (i.e. paper or article) was also categorised by the number of studies it contained. While a single publication represented one paper, it could include multiple studies if it examined more than one species, enrichment type, or experimental condition. For example, Tarou *et al.* (2004) included two separate studies on two species using different technological enrichments.

Enrichment types were categorised as sensory, food, cognitive, structural, or social, following the framework proposed by Young (2013). Since a single enrichment introduction could incorporate multiple types (e.g. a touchscreen device providing both cognitive and sensory stimulation), these were categorised accordingly.

Behavioural outcomes were classified as positive, negative, or neutral, based on the statistically significant behavioural changes and how they indicated improvements, detriments, or no significant impact on animal welfare. A single study could report multiple behavioural outcomes if different behavioural changes indicated both positive and negative effects. However, if multiple behavioural changes aligned within the same category (e.g. increased foraging, activity, and affiliative social behaviour, all considered positive indicators), they were recorded as a single outcome. A neutral behavioural outcome was assigned to studies that met inclusion criteria but reported no statistically significant changes in any measured behaviour. These outcomes reflect a lack of measurable effect based on the study’s analyses, regardless of whether animals visibly interacted with the enrichment.

This classification approach introduces a limitation, as behavioural outcomes rely upon the interpretation and measurement methods used in each study. The subjectivity inherent in these evaluations may affect the consistency and comparability of reported outcomes across studies. The data extraction form was piloted on a subset of studies and revised to ensure consistency and comprehensiveness. Disagreements during data extraction were resolved through discussion or consultation with a third reviewer. Authors of included studies were contacted for clarification or additional data when necessary.

### Data characterisation and synthesis

Extracted data were systematically organised in Excel® and visualised using GraphPad Prism 10 to identify trends and distributions. Data analysis included publication year, species studied, type of technology, enrichment type, interactivity level, and behavioural outcomes. Separate analyses were conducted for interactive and non-interactive technological enrichment. Technology types were further categorised by species to highlight usage differences across taxa.

The analysis was descriptive, in line with the objectives of a scoping review, and did not evaluate intervention effectiveness. Results were summarised using quantitative measures, including counts and percentages, and trends were illustrated using graphs and tables. This comprehensive methodology ensured a rigorous and systematic approach to exploring the landscape of technological enrichment in zoos, aquaria, and wildlife parks.

## Results

A total of 1,688 research articles were screened during the title and abstract review, with 181 proceeding to full-text screening. Of these, 22 articles were selected for data extraction, which comprised 25 studies due to multiple species tested within some publications (see *Data characterisation and synthesis* above). Figure 1 presents the PRISMA flow chart detailing the selection process. The studies collectively involved 123 individual animals (16 total species) across zoos (n = 18), aquaria (n = 2), and wildlife parks (n = 2). Most studies were physically conducted in the US (14 of 22 papers; 64%), followed by Europe (6 of 22; 28%), with one study each in Japan and Australia (4%). A summary of the relevant results has been provided in Table 1.

**Table 1.** Summary of the 22 articles (25 studies) included in the review of technological enrichment in captive animals. For each study, the table provides the species (with common and scientific names), sample size (n), enrichment type, type of technology used, behavioural outcome classification (positive, negative, or neutral), and the behaviour categories measured (e.g. active, stereotypies, social affiliative). Outcomes are based on statistically significant changes in behaviour.

Author(s)	Description	Species	Sample Size	Enrichment Type	Technology Type	Behavioural Outcome	Behaviour Class
Yanofsky & Markowitz (1978)	A push-button apparatus increased activity and foraging while reducing pacing and stationary behaviours in two mandrills but also resulted in notable declines in social and exploratory behaviours.	Mandrill (*Papio Sphinx*)	2	Structure; Food; Cognitive; Social	Push-Button	Positive Negative	Active, Inactive, Grooming, Social affiliative, Foraging, Stereotypies
Foster-Turley & Markowitz (1982)	The effects of a live prey-capture apparatus on the behaviour of captive otters. It indicated that the otters were highly motivated to hunt the crickets demonstrating an increase in activity and enclosure usage.	Asian Small-Clawed Otter (*Aonyx cinereum*)	7	Structure; Food; Sensory	Push-Button	Positive	Active
Carlstead *et* *al.* (1991)	Different feeding methods were tested on a black bear. Hiding food reduced stereotypic pacing, while the mechanical feeder showed less impact.	Black Bear (*Ursus americanus*)	1	Food	Response-Independent Feeder	Positive	Stereotypies, Foraging, Active
Markowitz *et* *al.* (1995)	An acoustic “prey” device was given to a captive leopard, finding that the device increased species-typical behaviours and decreased stereotypic behaviours.	African Leopard (*Panthera pardus*)	1	Structure; Food; Sensory	Motion Sensor	Positive	Active, Stereotypies
Tarou *et* *al.* (2004)	Two species of orangutans were given computer assisted tasks that resulted in an increase in aggression and scratching for both.	Sumatran Orangutan; Bornean Orangutan (*Pongo abelii; Pongo pygmaeus*)	5; 3	Cognitive; Sensory; Food	Computer	Negative; Negative	Stereotypies, Social affiliative, Social aggressive, Active
Wells *et* *al.* (2006)	Explored the impact of different types of auditory stimulation on the behaviour of gorillas. No significant effects were found.	Western Lowland Gorilla (*Gorilla gorilla gorilla*)	6	Sensory	Speaker	Neutral	Grooming, Social affiliative, Social aggressive, Active, Inactive, Stereotypies
Wells & Irwin (2008)	The impact of auditory stimulation on Asian elephants. The study found that the elephants displayed significantly less stereotypic behaviour when exposed to music compared to a control condition with no auditory stimulation.	Asian Elephant (*Elephas maximus*)	4	Sensory	Speaker	Positive	Active, Inactive, Social affiliative, Social aggressive, Stereotypies
Kistler *et* *al.* (2009)	Investigated the effects of feeding enrichment strategies on red foxes, finding that providing an electronic dispenser resulted in the highest levels of activity and food-related behaviours.	Red Fox (*Vulpes vulpes*)	4	Food	Response-Independent Feeder	Positive	Active, Inactive, Social affiliative, Social aggressive, Foraging
Perdue *et* *al.* (2012)	The influence of a touchscreen computer on orangutan behaviour and visibility. The study found that the touchscreen did not negatively affect orangutan behaviour.	Orangutan (unclear which species)	4	Cognitive; Sensory; Structure	Computer	Neutral	Social aggression, Stereotypies
Mallavarapu *et* *al.* (2013)	Computerised joystick enrichment was introduced to two species of orangutans. The enrichment decreased inactivity and scratching and increased feeding, but also led to elevated self-directed behaviours‥	Sumatran Orangutan; Bornean Orangutan (*Pongo abelii; Pongo pygmaeus*)	6; 2	Cognitive; Food	Computer	Positive Negative; Positive Negative	Active, Inactive, Social affiliative, Social aggressive, Stereotypies
Andrews & Ha (2014)	The effects of automated scatter feeders on grizzly bear behaviour, finding a significant decrease in time spent in repetitive behaviours and a significant increase in time spent active while the feeders were in use.	Brown Bear (*Ursus arctos*)	2	Food	Response-Independent Feeder	Positive	Active, Social affiliative, Social aggressive, Inactive, Stereotypies
Robbins & Margulis (2014)	Auditory enrichment, including music and natural sounds, resulted in a significant increase in hair-plucking in a gorilla, with no other behavioural changes reaching significance.	Western Lowland Gorllia (*Gorilla gorilla gorilla*)	4	Sensory	Speaker	Negative	Stereotypic, Active, Inactive, Foraging, Social affiliative, Social aggressive
Perdue (2016)	Investigated the effects of computerized testing on sun bear behaviour. The results indicated that the sun bears readily used a touchscreen computer, showed a preference for it over traditional enrichment. Though one subject did show an increase in pacing.	Sun Bear (*Helarctos malayanus*)	2	Cognitive; Sensory; Structure	Computer	Positive Negative	Social aggressive, Stereotypies
Winship & Eskelinen (2018)	Novel video clips were presented to two dolphin species. While some animals investigated the screen, male dolphins showed increased aggression toward the television.	Rough-Toothed Dolphin; Bottlenose Dolphin (*Steno bredanensis; Tursiops truncatus*)	5; 12	Sensory; Social	Video	Positive Negative; Positive	Active, Social aggressive
Grunauer & Walguarnery (2018)	The effects of the use of a painting application on a digital tablet device on chimpanzees. The study found that the digital tablet was effective in reducing some stereotypic and displacement behaviours.	Chimpanzee (*Pan troglodytes*)	8	Sensory; Structure; Cognitive	Computer	Positive	Stereotypies, Inactive, Active
Jacobson *et* *al.* (2019)	Examined the effects of giving macaques computer-based tasks on their welfare by observing their behaviour during times when the computer was available and when it was not. The study found that while most behaviours did not differ significantly between conditions, there was a significant increase in aggression observed during the computer sessions.	Japanese Macaque (*Macaca fuscata*)	12	Cognitive; Sensory	Computer	Negative	Grooming, Social affiliative, Social aggressive, Stereotypies, Active, Inactive
Garcia *et* *al.* (2020)	Scheduled visual enrichment increased activity and reduced cortisol in a captive orangutan, suggesting improved well-being; but also led to an increase in scratching behaviour.	Bornean Orangutan (*Pongo pygmaeus*)	1	Sensory	Video	Positive Negative	Active, Inactive, Foraging, Grooming
Carter *et* *al.* (2021)	Evaluated a digital enrichment system using interactive projections for orangutans. It did not significantly alter most of their observed behaviours.	Sumatran Orangutan (*Pongo abelii*)	5	Structure; Sensory	Projector	Neutral	Active. Inactive, Foraging, Stereotypies
Hirskyj-Douglas & Kankaanpää (2021)	Explored how white-faced sakis would choose to control a digital visual enrichment system by having the ability to turn on the videos through a motion sensor. They found that the visual stimuli reduced the sakis’ scratching behaviour.	White-Faced Saki (*Pithecia Pithecia*)	7	Sensory; Structure; Social	Video	Positive	Social affiliative, Social aggressive, Inactive, Active, Stereotypies
Guérineau *et* *al.* (2022)	Evaluated the effects of music as an environmental enrichment for bottlenose dolphins, finding that it increased several social affiliative behaviours during and after its presentation.	Bottlenose Dolphin (*Tursiops truncatus*)	8	Sensory	Speaker	Positive	Social affiliative, Inactive, Social aggressive, Active
Caselli *et* *al.* (2022)	Auditory enrichment using territorial call playback reduced stress-related behaviours in ring-tailed lemurs;but also increased agnostic behaviours.	Ring-Tailed Lemur (*Lemur catta*)	6	Sensory; Social	Speaker	Positive Negative	Active, Inactive, Foraging, Stereotypies, Social affiliative, Social aggressive, Grooming
Yamanashi *et* *al.* (2022)	Investigated the use of interactive movie art as a form of environmental enrichment for chimpanzees, finding that the chimpanzees spent more time in the indoor enclosure when the interactive art was present and that younger chimpanzees showed positive responses to the art.	Chimpanzee (*Pan troglodytes*)	6	Sensory; Cognitive	Motion Sensor	Positive	Inactive, Foraging, Active, Grooming, Social affiliative, Social aggressive, Stereotypies

*Note:* Papers that have two studies also have sample sizes and behavioural outcomes listed per study (separated by semicolons)

**Figure 2.**
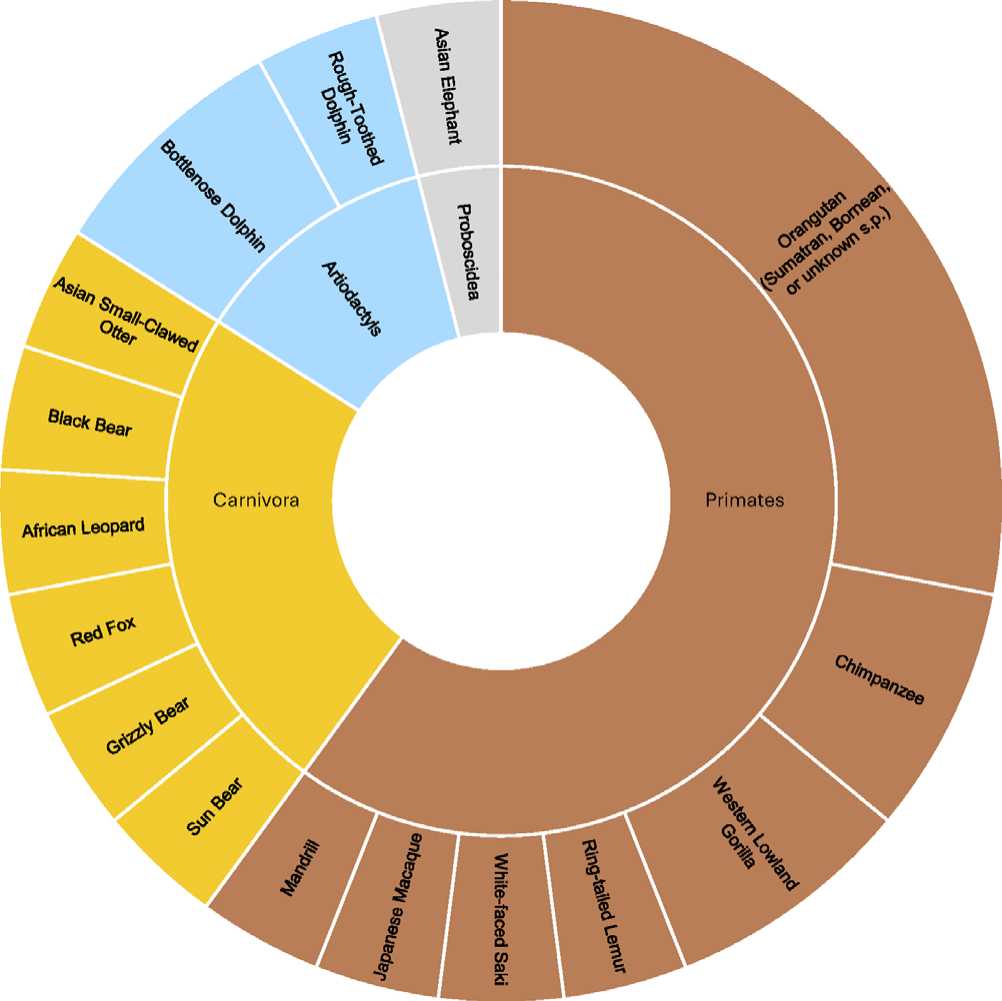
Distribution of species used in technological enrichment studies, grouped by taxonomic order. The sunburst chart illustrates the number of studies associated with each species (e.g. orangutan [*Pongo* spp], chimpanzee [*Pan troglodytes*], sun bear [*Helarctos malayanus*]) and higher taxonomic grouping (e.g. primates, carnivores). Larger segments represent species more frequently studied.

Although Table 1 provides a summary of species representation, Figure 2 visually highlights the distribution of species studied in technological enrichment research. The studies encompassed a range of species, primarily focusing on primates (n = 15), followed by carnivores (n = 6), artiodactyls (n = 3), and one proboscidean. Orangutans (*Pongo* spp) were the most studied species (n = 7), followed by chimpanzees (*Pan troglodytes*) (n = 2), western lowland gorillas (*Gorilla gorilla gorilla*) (n = 2), and bottlenose dolphins (*Tursiops truncatus*) (n = 2). Other species included the rough-toothed dolphin (*Steno bredanensis)* (n = 1), Asian elephant (*Elephas maximus*) (n = 1), sun bear (n = 1), and African leopard (n = 1).

**Figure 3.**
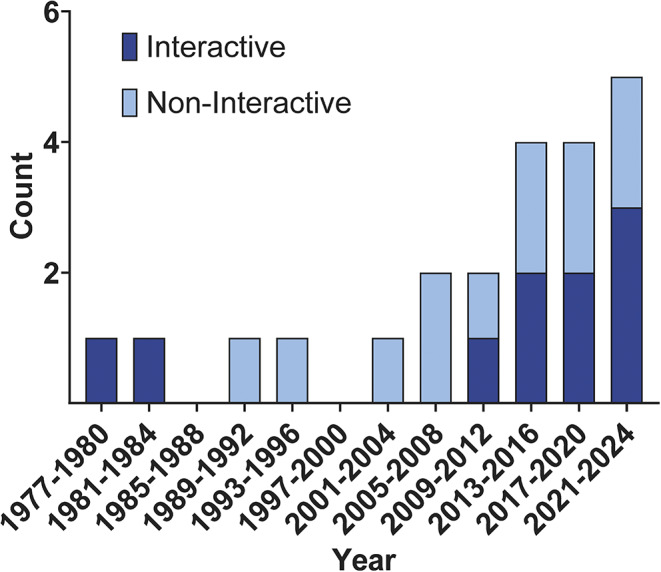
Number of articles published from 1978 to 2024 that assessed technological enrichment in zoos, aquaria, or wildlife parks. The dark blue bar shows the total number of articles by year, while the light blue bar shows the subset of studies involving interactive technologies (e.g. touchscreen devices or motion sensors).

Technological enrichment publications began in 1978 with the earliest research involving push-button enrichment for mandrills (*Papio sphinx*) (Yanofsky & Markowitz 1978). Since the first publication in 1978, research on technological enrichment has been sporadic, with minimal output between 1996 and 2003. A slight increase in interest is evident starting in 2004, peaking in 2022 with three publications. A notable rise in interactive technological enrichment studies occurred in 2012, with the highest number of such studies being two in 2021. These trends are illustrated in Figure 3. Most articles were published in *Zoo Biology* (n = 8), followed by *Applied Animal Behaviour Science* (n = 4), *Animal Welfare* (n = 3), and *Animals* (n = 2), with the remainder published in various other journals.

**Figure 4.**
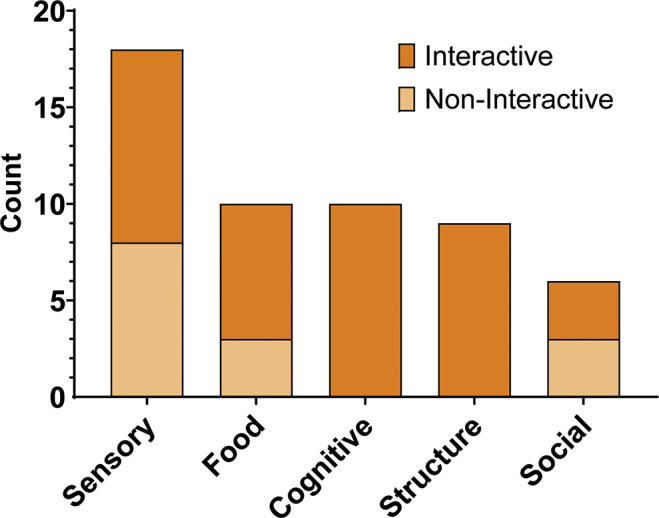
Frequency of each enrichment type (sensory, food, cognitive, structural, social) across the 25 studies reviewed. Bars are divided by interactivity: dark orange = interactive technology (e.g. touchscreens, push-buttons), light orange = non-interactive technology (e.g. speakers, automated feeders).

All studies involved enrichment categorised as sensory, food, cognitive, structural, or social, with each enrichment able to consist of multiple types simultaneously (a total of 53 types of enrichment across the 25 studies; see Figure 4). These 25 studies were drawn from 22 peer-reviewed articles, with some articles reporting more than one study (e.g. testing multiple species or enrichment types). Sensory enrichment was the most frequent (n = 18), including examples such as auditory stimuli (e.g. music, animal vocalisations) and visual media (e.g. video clips or projections). Cognitive enrichment (n = 10) often involved problem-solving or computer-based tasks such as touchscreens or joysticks. Food enrichment (n = 10) included devices that dispensed food either automatically (e.g. response-independent feeders) or through animal interaction (e.g. push-buttons or prey-releasing systems). Structural enrichment (n = 9) referred to physical or spatial alterations, such as light projections or apparatus that could be activated or manipulated. Social enrichment (n = 6) included digital stimuli that mimicked social interaction, such as video playbacks of conspecifics or interactive media involving group dynamics.

Interactive enrichment dominated cognitive and structural enrichment types (both 100%), while sensory enrichment included both interactive (n = 10) and non-interactive (n = 8) elements. Food enrichment was also split, with interactive (n = 7) and non-interactive (n = 3) instances. Additionally, social enrichment showed an even distribution between interactive (n = 3) and non-interactive (n = 3) approaches.

Technological types were classified into interactive and non-interactive categories (Table 2). Interactive technologies (n = 13) included computers (n = 8), motion sensors (n = 2), projectors (n = 1), and push-buttons (n = 2). Non-interactive technologies comprised speakers (n = 5), videos (n = 4), and response-independent feeders (n = 3). Many interactive technologies were given to primates, whereas non-interactive technological enrichment had a more diverse species range.

**Table 2.** Classification of technological enrichment types used in zoo, aquaria, and wildlife park studies. Technologies are categorised as interactive or non-interactive, with definitions, the number of studies using each technology, and the species (common names) involved. Interactive technologies require animal-initiated input (e.g.computers, push-buttons), whereas non-interactive devices operate automatically (e.g., speakers, response-independent feeders)

Interactive		Definition	Count	Species
	Computer	Electronic devices equipped with screens and capable of running programs, displaying content, or enabling interactive experiences	8	Orangutan, Chimpanzee, Sun Bear, Japanese Macaque
	Motion Sensor	Device that detects the presence or movements of animals and responds accordingly	2	African Leopard, Chimpanzee
	Projector	Device that displays visual content onto surfaces within the animal’s enclosure	1	Orangutan
	Push-button	A mechanical or digital button that triggers a pre-programmed response when pressed by the animal	2	Mandrill, Asian Small-Clawed Otter

Non-Interactive				
	Speaker	Device that plays audio content, such as recorded animal calls, music, or environmental sounds	5	Gorilla, Elephant, Lemur, Dolphin
	Video	Moving images or pre-recorded content to stimulate animals visually	4	Dolphin, Orangutan, White-Faced Saki
	Response Independent Feeder	Device that releases food or treats at predetermined intervals	3	Red Fox, Black Bear, Grizzly Bear

**Figure 5.**
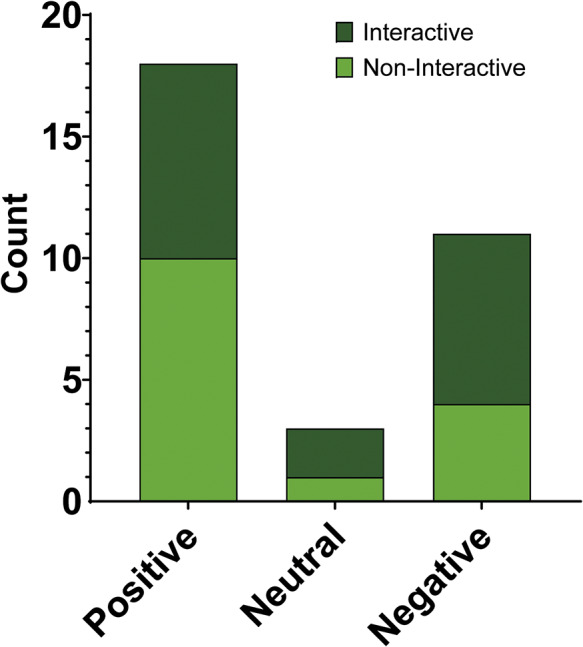
Distribution of behavioural outcome classifications across the 25 studies reviewed. Bars represent the number of studies reporting positive, negative, or neutral effects on behaviour. Each bar is divided by technology type: dark green = interactive technology; light green = non-interactive technology.

Behavioural outcomes were classified as positive, negative, or neutral, based on a statistically significant welfare effect observed for at least one measure in a study. Studies were categorised as neutral when no significant behavioural changes were found. In these cases, the technological enrichment met all inclusion criteria and was implemented as intended but failed to elicit a measurable effect on the behaviours assessed. This does not necessarily imply the enrichment had no value, but rather that no observable change occurred within the parameters measured. Positive outcomes were the most common (n = 18 effects across 25 studies), with eight from interactive enrichments. Negative outcomes totalled eleven effects across 25 studies, primarily from interactive enrichments (n = 7). Neutral outcomes were observed in three cases, distributed across interactive (n = 2) and non-interactive (n = 1) enrichments. For the welfare outcomes across each category, see Figure 5.

Outcome distribution also varied by taxonomic group. Most outcomes were reported in studies involving primates (n = 20 primate outcome effects), including all three neutral outcomes and the majority of negative ones (nine of eleven total negative outcomes; 82%). Only eight of the 20 primate outcome effects (40%) were positive. Carnivore studies accounted for seven outcomes, with six positive (86%) and one negative outcome (14%). Studies involving dolphins accounted for four outcomes, with three positive (75%) and one negative (25%). There was one proboscidean study outcome done with Asian elephants, which was positive. These trends should be interpreted in relation to the number of studies conducted per group, as primates were the most frequently studied taxa.

All studies examined at least one behavioural category: social behaviour (n = 18), active behaviour (n = 16), abnormal behaviour (n = 15), inactivity (n = 10), or aggression (n = 8). The majority of studies (23 of 25; 92%) conducted pre/post analyses, comparing baseline and treatment phases within subjects, while eight studies (32%) used comparisons between subjects. Six of the studies (24%) employed both approaches. Only one study incorporated a physiological measurement (the stress hormone, cortisol) alongside behavioural observations, with the remaining studies relying exclusively on behavioural data. The physiological data showed a decrease in salivary cortisol when given the enrichment (Garcia *et al.* 2020).

## Discussion

Our review examined the use of technological enrichment in zoos, aquaria, and wildlife parks identifying trends in species representation, enrichment type, and reported behavioural outcomes. Findings highlight both the growing interest in technological enrichment and the methodological gaps that must be addressed to evaluate its effectiveness systematically.

### Prevalence of interactive technologies

Interactive technologies, such as computers, push-buttons, and motion sensors, are widely studied (Foster-Turley & Markowitz 1982; Grunauer & Walguarnery 2018; Jacobson *et al.* 2019; Yamanashi *et al.* 2022). These systems engage animals in sensory, cognitive, and structural enrichment. However, sensory, food, and social enrichment types include both interactive and non-interactive elements, demonstrating the continued role of passive enrichment (Kistler *et al.* 2009; Robbins & Margulis 2014; Yamanashi *et al.* 2022). Effectiveness depends upon species and intended welfare outcomes. Further research should explore how choice, control, novelty, and cognitive challenge influence enrichment benefits.

### Growth of technological enrichment research

Technological enrichment studies have grown steadily, particularly since the early 2000s. The increase in studies since the first interactive study in 1978, with a peak in 2022, reflects a rising interest in using technology for animal welfare (Yanofsky & Markowitz 1978; Tarou *et al.* 2004; Perdue *et al.* 2012; Hirskyj-Douglas & Kankaanpää 2021). However, despite this growth, the overall number of publications remains low, with a peak of only three papers in a single year and a total of 22 articles meeting this review’s criteria. As this review focused exclusively on studies that included a direct behavioural comparison (e.g. pre/post or control vs enrichment), only a small subset of the broader literature met the inclusion criteria. While the field is expanding, it remains a niche research area. The limited focus on certain technologies, such as touchscreens, may be due to their relatively recent accessibility, influenced by the widespread adoption of computers, reduced technology costs, and insights from cognitive research with primates.

### Species representation and bias

This review highlights a lack of species diversity in technological enrichment studies, with a strong bias toward primates, particularly great apes. This emphasis may be influenced by historical research patterns, their frequent use in cognitive and behavioural studies, and the practical availability of computer-based tools suited to their abilities (Melfi 2005; Hopper 2017). Additionally, the prevalence of computer-based technologies may be linked to the assumption that species with manual dexterity, such as primates, are best suited to interact with these devices. Historical precedence in studying primates in cognitive and behavioural contexts has further reinforced their dominance in technological enrichment research (Binding *et al.* 2020; Williams *et al.* 2023).

Expanding research beyond primates to a broader range of taxa, including non-primate mammals, birds, reptiles, and amphibians, would improve the generalisability of findings and ensure enrichment strategies cater to diverse species-specific needs. Some studies suggest that non-primate species can interact with technology in creative ways, such as sun bears using their tongues to engage with touchscreens (Perdue 2016). These findings highlight the potential for technological enrichment to be adapted for species without manual dexterity, emphasising the need for further research into its applications across taxa.

In addition to being overrepresented in the literature, primates also accounted for the majority of neutral and negative outcomes observed in this review. Of the 20 total outcomes reported in primate studies, nine were negative and three were neutral. In contrast, studies involving carnivores and artiodactyls reported primarily positive outcomes. While these differences must be interpreted cautiously due to the small number of studies per group, they may reflect underlying differences in enrichment types used, cognitive demands of the enrichment, or methodological expectations. These findings underscore the importance of tailoring technological enrichment to the needs and abilities of each species, and they highlight the potential for comparative, cross-taxa research to reveal more nuanced insights into enrichment effectiveness.

### Trends in enrichment types

Sensory enrichment emerged as the most common type of technological enrichment, with audio or visual stimuli from speakers or videos frequently used in both interactive and non-interactive forms (Wells & Irwin 2008; Winship & Eskelinen 2018; Hirskyj-Douglas & Kankaanpää 2021; Guérineau *et al.* 2022). Although sensory enrichment is well represented, other types, such as structural and social enrichment, are less commonly studied with technological enrichment, pointing to an imbalance in the exploration of different enrichment types (Yanofsky & Markowitz 1978; Perdue *et al.* 2012; Grunauer & Walguarnery 2018; Carter *et al.* 2021; Caselli *et al.* 2022).

The prevalence of sensory enrichment may be due to its relative ease of implementation compared to other enrichment types. Playing music or bird sounds requires fewer resources and less logistical effort than integrating technology into structural designs or creating interactive systems. This may explain why structural and social enrichment are underrepresented despite their potential welfare benefits. Future strategies, such as Temporary Exhibit Design (TED; Fernandez *et al.* 2023), may offer a framework for more flexible and adaptive integration of technological elements into exhibit structures, facilitating research and implementation of structural technological enrichment.

### Behavioural outcomes and methodological challenges

The behavioural outcomes of technological enrichment have been largely positive, with most studies reporting beneficial changes. Both interactive and non-interactive enrichment produced favourable effects in similar proportions, indicating that interactive technologies can successfully promote positive behaviours in captive animals. However, negative and neutral outcomes have also been observed, particularly when undesirable behaviours, such as stereotypies, increased in response to enrichment (see Table 1). Although these findings provide insights into enrichment effects, they rely solely upon behavioural changes as welfare proxies, which may not fully capture long-term welfare states. Short-term behavioural variations may reflect immediate responses rather than sustained improvements, emphasising the need for longitudinal studies to determine the persistence of these effects. Additionally, neutral outcomes, such as unchanged activity levels, should be interpreted with caution, as they may not indicate welfare improvements (see Table 1).

Methodologically, behavioural observations dominated assessments, with 84% of studies using ethograms. In contrast, physiological measures were rarely utilised, with only one study incorporating salivary cortisol to assess stress or welfare (Garcia *et al.* 2020). This represents a significant limitation, as behavioural data alone may not fully capture enrichment impacts. Many studies also focused on short-term behavioural responses, leaving gaps in understanding long-term engagement and potential habituation effects. The logistical and financial challenges of implementing physiological measures in zoo settings may explain their limited use (de Azevedo *et al.* 2007; Clay *et al.* 2011; Rose & Riley 2021). Despite these constraints, future research should integrate both behavioural and physiological assessments to provide a more comprehensive evaluation of technological enrichment’s effects on welfare.

### Publication bias and study limitations

The limited number of studies identified in this review and the predominance of positive findings suggest potential publication bias, where studies reporting no effect or negative outcomes may be underrepresented. These findings highlight the importance of not assuming enrichment will be effective simply by virtue of being novel or technological. Neutral outcomes suggest a need to better understand which types of enrichment align with species-specific needs and underscore the value of publishing non-significant results to avoid publication bias in welfare research. Additionally, methodological inconsistencies, including small sample sizes and lack of experimental controls, hinder direct comparisons across studies. Establishing standardised methodologies for assessing technological enrichment — such as structured observation protocols and clear welfare indicators — will be essential for future progress.

Although several innovative technologies for animal engagement are reported in the Animal-Computer Interaction (ACI) and Animal-Centred Computing (ACC) literature, these studies often focus on usability, interaction feasibility, or task-solving rather than on changes in behavioural indicators relevant to welfare. As such, they were excluded from this review, which specifically focused on studies that reported behavioural outcomes across at least two conditions. Nonetheless, the development of such technologies plays an important role in advancing enrichment design, and future reviews may benefit from integrating these perspectives.

Although this review applied a behavioural and welfare-focused operational definition of *interactive* (requiring observable animal manipulation resulting in a direct consequence), we acknowledge that interactivity is a broader and more nuanced concept within HCI and ACI literature. For example, systems that respond to passive presence, unintentional triggering, or autonomous sensing without overt animal engagement may also be described as interactive, particularly in the context of interface design or user experience. Although such systems were beyond the scope of this review, which required comparative behavioural outcome data, they represent valuable directions for innovation and have been explored in studies identified during screening. Many of these studies were authored by multidisciplinary teams, including researchers from ACI, HCI, and animal welfare, such as Grant *et al.* (2023), Kleinberger *et al.* (2023, 2024), and French *et al.* (2018, 2020, 2022). Future work may benefit from integrating these perspectives to explore how definitions of interactivity differ across disciplines and how design-centred approaches can complement welfare-focused evaluation.

### Future directions

Future studies should expand beyond primates and incorporate more non-interactive technologies, particularly those aligned with species-specific behaviours. Longitudinal studies should examine how animals engage with these technologies over time to assess habituation effects and long-term welfare impacts (Kuczaj *et al.* 2002). Addressing financial and logistical constraints will be critical to ensuring the broader implementation of technological enrichment in zoos and aquaria.

### Animal welfare implications

The findings of this review suggest that technological enrichment can positively impact animal welfare by promoting engagement, cognitive stimulation, and species-appropriate behaviours. Both interactive and non-interactive technologies contribute to enrichment programmes, although research remains biased toward primates and certain sensory-based technologies. Expanding technological enrichment to a broader range of species and incorporating standardised welfare assessments, including physiological measures, will be critical to understanding its long-term benefits. Addressing current methodological gaps and species biases will help ensure that technological enrichment is used effectively to enhance the well-being of animals in human care.

## Conclusion

Technological enrichment has garnered growing interest in recent years, though several critical gaps remain in our understanding of its effectiveness. While zoos, aquaria, and wildlife parks have begun implementing various forms of technological enrichment, there is a lack of peer-reviewed studies supporting these practices. To move the field forward, research must not only expand to include a wider range of species but also focus on more comprehensive and quantifiable measurements of the use and impact of technological enrichment. This will allow for a clearer understanding of its effects on animal behaviour and welfare, particularly the long-term impacts of such enrichment. As with any form of enrichment, the novelty effect should be considered, and future studies must investigate whether behavioural changes are sustained over time or if habituation occurs, potentially diminishing the effectiveness of the enrichment.

In particular, there is a need to explore how incorporating interactive components into technological enrichment might enhance its effectiveness. The potential benefits of offering animals choice should be considered, as previous research has shown that such autonomy may positively influence welfare outcomes (Rust *et al.* 2024). However, biases in the application of technological enrichment, such as a possible focus on primates, should be examined critically to ensure a broader, more inclusive approach to enrichment practices. Future research could explore how the design of technological enrichment systems impacts various species, considering the effects of novelty and habituation, and assess whether biases influence how different animal groups are enriched.

Ultimately, further research is needed to guide the development of evidence-based practices, better understand the nuances of technological enrichment, and ensure that these tools are being used in ways that genuinely benefit animal welfare over both the short and long term.
